# A Case of Fatal Abscess of the Kidney

**Published:** 1840-01

**Authors:** L. Powell

**Affiliations:** Louisville


Art. II.—*Case of Fatal Abscess of the Right Kidney.* By L. Powell, M. D. of Louisville.

Mr. B. S., ætat. 30, of tall stature, spare habit, and irritable temperament, had for several years been more or less affected with a sense of weight and fulness, with a feeling of uneasiness or obscure pain in the lumbar region, accompanied with an irritable state of the bladder, the urine being habitually discharged at short intervals, in small quantity and of unnatural color and consistence, being always more or less turbid, sometimes milky and frequently of brownish red color and depositing on standing more or less sediment.

When consulted in his case, and informed of the symptoms just detailed, I could not fail to infer a serious irritation or perhaps a subacute inflammation of the kidneys to be its true diagnostic character or the real pathological condition.

In accordance with this view he was restricted of exercise, confined to a strict anti-phlogistic regimen, subjected to one general bleeding and to frequent cuppings over the loins, to the moderate use of laxative medicines and demulcent drinks, and finally to counter irritation by a seton inserted in the back and worn for several months. Under this course of treatment the symptoms were palliated, and the comfort of the patient improved. It was obvious, however that no permanent benefit had accrued, and in the vague hope that travel and mineral waters might restore his health, the unfortunate gentleman visited the most celebrated springs of Virginia, made frequent and extensive excursions through the country, and sojourned one winter in the south, by which time a very sensible impairment of his general health had succeeded to the local symptoms. He returned to Louisville late in the

Fall of 1837, and again called my attention to his condition. He was now much reduced in flesh and strength, his complexion very sallow and the whole skin unnaturally dry and harsh, while the cuticle on the palms of the hands and the soles of the feet was exceedingly thickened and marked by fissures or cracks. But the most striking feature of the case in its present aspect was a large abdominal tumour occupying the right iliac and right hypochondriac regions and extending laterally in the opposite direction to a short space beyond the umbilicus. The tumour was in shape an irregular oblong of uniform surface, exceedingly tense and rather unyielding but slightly elastic under pressure. In reference to the further treatment of the case it was of the first importance that the nature and origin of this tumour should be satisfactorily ascertained. The difficulty of this task, however, was not a little increased by the various and discrepant opinions which had been passed upon it by the several medical gentlemen whom the patient had consulted at different points. Looking to the situation of the tumour, and its apparent connection with the symptoms of renal inflammation which had so long and so constantly preceded it, I felt myself justified in the decision that it resulted from and consisted in some morbid growth or structural change in the kidney itself.

After repeated examinations of the tumour, in which I was accompanied and aided by Dr. Flint, professor of Surgery in the Louisville Medical Institute, we thought that fluctuation was perceptible, and that the case in its present stage might be considered and treated as Abscess of the Kidney.

We accordingly made an incision into the tumour, when purulent matter of ordinary consistence and of greenish yellow color issued from the orifice, and continued to flow until full half a gallon had been collected in the vessel which received it. A few days after the orifice having closed was again opened, when an additional quantity of pus was discharged, amounting perhaps to about one quart. From this time the orifice remained open, and the matter continued to be spontaneously discharged from day to day, in various quantity. For several weeks the case wore a most favorable aspect; no constitutional disturbance succeeded to the operation: the patient became cheerful in the hope of recovery, slept soundly, ate and digested a fair allowance of food, and enjoyed a regular state of the bowels, and an improved condition of the surface. At about four weeks from the date of the operation upon the tumour, and after an anxious and sleepless night, consequent upon an important transaction of business in which he had been involved, he was seized with a strong rigor, to which ensued in regular succession, and by daily paroxysms, a well defined hectic fever, bringing in its train cough, night-sweats, colliquative diarrhœa, which terminated his life about ninety days from the opening of the abscess.

A post mortem examination exhibited the following circumstances:—The abdominal points were divided by an incision over the tumour extending from the sternum to the pubis, by which the tumour, consisting now of the half empty sack of the abscess, was brought into view, extending from the natural situation of the kidney below, to the concave surface of the liver above, and united by old and strong adhesions, to all the contiguous parts and organs, to the walls of the abdomen, the folds of the intestines, and the concave face of the liver—where the union was so extensive and intimate as to be inseparable by the knife, seeming to constitute, with the liver, a continuous and identical structure of morbid condition and aspect. On enlarging the opening in the sac, and sponging out the matter which it contained, no trace of the natural structure of the kidney remained; the substance of that organ seemed to have been utterly disorganized and destroyed, leaving only its original tunic now converted by inflammation and pressure into the diseased, thick walls of the abscess.

On exploring the cavity of the abscess, it was discovered to be divided into several compartments or pockets, in each of which was contained a quantity of urinary calculi of various sizes, from a millet-seed to the largest garden pea, and so lodged and compacted in these pouches, that they could not find their way into the general cavity of the abscess, while on the other hand, they could not be carried forward to the bladder—the renal orifice of the ureter having been completely obliterated by inflammation. The kidney of the opposite side was of normal structure, but rather paler than natural, and enlarged considerably beyond its usual size, and containing in its pelvis a single calculus of a soft and firiable texture, marking the incipient stage of a similar disease in the left kidney, to that which had already destroyed the right. The dissection was not extended to the lungs or other organs, in consequence of the intention of the friends of the deceased to convey his body for interment to a distant county. Enough, however, had been revealed to furnish to me the satisfactory assurance, that I had not erred in the diagnosis of the case, and to convey to his friends, the melancholy conviction that no course of treatment could have conducted it to a different issue.

